# Endoscopic transnasal approach to pterygopalatine fossa and infratemporal fossa: case series utilizing endoscopic modified medial maxillectomy (EMMM)/direct approach to the anterior and lateral part of the maxillary sinus (DALMA)

**DOI:** 10.1007/s00701-026-06837-z

**Published:** 2026-03-17

**Authors:** Motoyuki Umekawa, Hirotaka Hasegawa, Yuki Shinya, Masahiro Shin, Kenji Kondo, Satoru Miyawaki, Nobuhito Saito

**Affiliations:** 1 Department of Neurosurgery, The University of Tokyo Hospital, Tokyo, Japan; 2 Department of Neurosurgery, Saitama Medical Center, Saitama Medical University, Kawagoe, Saitama Japan; 3 Department of Neurosurgery, University of Virginia, Charlottesville, VA USA; 4 Department of Neurosurgery, Teikyo University Hospital, Tokyo, Japan; 5 Department of Otorhinolaryngology and Head and Neck Surgery, The University of Tokyo Hospital, Tokyo, Japan

**Keywords:** Endoscopic transnasal surgery, Skull base, Pterygopalatine fossa, Infra-temporal fossa, Endoscopic modified medial maxillectomy, Direct approach to the anterior and lateral part of the maxillary sinus

## Abstract

**Background:**

Endoscopic transnasal surgery (ETS) has become an established treatment for skull base lesions; however, its lateral reach to the pterygopalatine fossa (PPF) and infratemporal fossa (ITF) remains limited due to restricted maneuverability within the nasal corridor. To address this limitation, the endoscopic modified medial maxillectomy (EMMM) and the direct approach to the anterior and lateral part of the maxillary sinus (DALMA) provides lateral access through the maxillary sinus. This study evaluated the technical feasibility and outcomes of ETS combining EMMM and DALMA.

**Method:**

A retrospective review was conducted on five patients (January 2021–April 2024) who underwent ETS involving EMMM and/or DALMA. Surgical trajectory, extent of resection, and postoperative outcomes were analyzed. EMMM preserved nasolacrimal duct and opened the medial maxillary wall for direct access to the PPF and ITF. In DALMA, submucosal dissection was extended laterally beyond the piriform aperture to remove the anterior maxillary wall creating as an additional port complementary to EMMM.

**Results:**

Two patients (trigeminal schwannoma, idiopathic cerebrospinal fluid leak) underwent EMMM alone, and three patients (meningiomas extending to the ITF/PPF) underwent combined EMMM and DALMA. The dual-portal configuration provided separate working corridors for the endoscope and instruments. This arrangement minimized mutual interference and improved surgical maneuverability, allowing exposure of the lateral skull base up to the medial border of the mandible. Gross or subtotal resection was achieved in all cases. Postoperative complications were minimal; one patient experienced slight worsening of facial numbness, and one experienced slight numbness in the upper lip, likely due to DALMA-induced injury of the anterior superior alveolar nerve.

**Conclusions:**

Combining EMMM and DALMA approaches provides a safe and minimally invasive option for treating lesions extending laterally into the ITF and PPF. Dual-portal configuration appears to improve instrument maneuverability and facilitate more extensive resection, thereby potentially expanding the applicability of ETS.

**Supplementary Information:**

The online version contains supplementary material available at 10.1007/s00701-026-06837-z.

## Introduction

Pterygopalatine fossa (PPF) and infratemporal fossa (ITF) are complex regions of the skull base that are difficult to access but are occasionally invaded by tumors such as craniofacial meningiomas, schwannomas, and skull base malignancies [22]. Traditionally, these lesions necessitated craniofacial approaches involving extensive craniotomies and/or maxillary mobilization [1, 16]. However, given that such approaches are highly invasive, particularly for benign lesions, minimally invasive approaches are preferred from a standpoint of functional preservation and cosmetic outcomes. Endoscopic transnasal surgery (ETS) has been established as an effective minimally invasive technique for midline and paramidline skull base lesions [3, 4, 9]. Nonetheless, ETS generally offers limited access and restricted maneuverability when addressing laterally extending pathologies in such as those involving the ITF and PPF.

In ETS, direct access to the ITF and PPF requires traversing the maxillary sinus. The endoscopic modified medial maxillectomy (EMMM) is known to be one of the representative approaches to access the maxillary sinus from the anteromedial aspect while preserving the nasolacrimal duct and inferior turbinate. However, EMMM has been mainly utilized for rhinological procedures, and reports on EMMM for neurosurgical pathologies remain limited [17, 24]. Thus, its efficacy in neurosurgical procedures remains to be elucidated. Additionally, we have recently employed another method to access the maxillary sinus, called the direct approach to the anterior and lateral part of the maxillary sinus (DALMA), which allows for a more lateral access to the ITF and PPF via an endonasal route outside the piriform aperture [18]. Importantly, DALMA can be used in combination to EMMM, allowing for multiportal access to the ITF and PPF, leading to improved maneuverability. Herein, we report our preliminary experience with EMMM and DALMA for ITF and PPF lesions.

## Method

From January 2021 to April 2024, we retrospectively extracted patient data from our institutional database of 199 ETS procedures involving either EMMM or DALMA. Patient background, pathology, surgical approach, postoperative outcomes, and complications were evaluated. All participants provided written informed consent, and the study was approved by the Institutional Review Board of The University of Tokyo Hospital (IRB number: 2231).

### Surgical setting

**Fig. 1:**
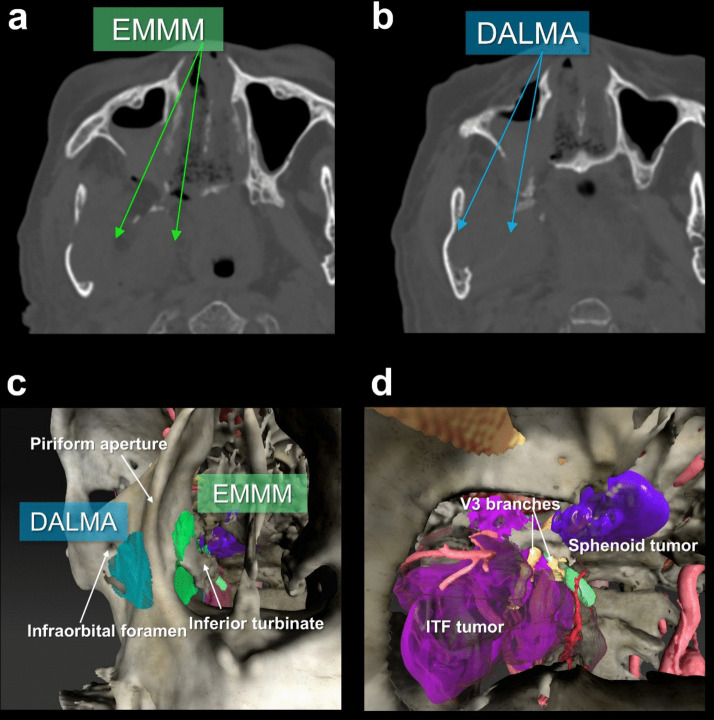
Schematic illustration of the approach routes of endoscopic modified medial maxillectomy (EMMM) and the direct approach to the anterior and lateral part of the maxillary sinus (DALMA) to the right pterygopalatine fossa and infratemporal fossa. Postoperative CT images of Case 3 demonstrate the extent of medial maxillary wall drilled by EMMM **(a)** and anterior maxillary wall drilled by DALMA **(b)**. Three-dimensional reconstruction images show operative windows of EMMM** (c, green area)** and DALMA **(c. blue area)**, and operative view via DALMA corridor **(d)**

All procedures were performed under general anesthesia with the patient in a supine position, head elevated approximately 15 degrees and fixed in a Mayfield head holder in a neutral rotation. Nasal decongestion was achieved using epinephrine-soaked pledgets. For tumor components involving the ITF and PPF, a uninostril approach was utilized to perform EMMM and/or DALMA (Fig. 1a). These approaches were typically combined with a binostril approach to facilitate bimanual manipulation and access to deeper midline structures such as skull base adjacent to the internal carotid artery, clivus, and sella. Rigid endoscopes (4 mm, 0°, 30°, and 70°) were used and stabilized with a robotic holding arm. Bimanual manipulation was typically performed using a bendable long-shaft bipolar forceps designed for endonasal skull base surgery (Fujita Medical Instrument, Tokyo, Japan), high-speed drill, or an ultrasonic aspirator in the dominant hand and an irrigation suction device in the nondominant hand. The working side was selected according to tumor laterality. When operating through the right nasal cavity, the dominant right-hand instrument was introduced via the EMMM corridor, whereas the endoscope and nondominant-hand instrument shared the DALMA corridor. Conversely, when operating through the left nasal cavity, the configuration was mirrored. Although instruments converge at the nostril entry, spatial separation was achieved within the maxillary sinus, where the EMMM and DALMA windows created distinct internal trajectories. A contralateral transseptal corridor could also be utilized when additional maneuverability was required. Neuronavigation and neurophysiological monitoring were employed to enhance intraoperative safety. During skull base procedures, irrigation suction was routinely utilized for intermittent irrigation to prevent thermal injury and maintain clear visualization. A drill with integrated irrigation and an ultrasonic aspirator providing continuous irrigation were also used during resection. When high-flow cerebrospinal fluid leakage was anticipated, a lumbar drainage system with a pressure-control valve was placed postoperatively.

### Endoscopic modified medial maxillectomy (EMMM)

The mucosa of the prelacrimal lateral nasal wall was incised and dissected from the submucosal margin of the inferior turbinate to the inferior edge of the piriform aperture. The nasolacrimal duct was identified and meticulously preserved. The bone overlying the nasolacrimal duct was then removed using high-speed drill and/or Kerrison punches to medialize the nasolacrimal duct and inferior turbinate. The medial wall of the maxillary sinus was then widely removed from the prelacrimal space all the way back to the PPF, allowing for wide exposure of the posterior wall of the maxillary sinus (Fig. 1b).

### Direct approach to the anterior and lateral part of the maxillary sinus (DALMA)

Through the mucosal incision made for the EMMM, the piriform aperture was identified, and submucosal dissection was extended laterally to expose the anterior wall of the maxillary sinus. The lateral limit was the infraorbital foramen through which the infraorbital nerve passes. Of note, DALMA may result in injury to the anterior superior alveolar nerve (ASAN), which lies in close proximity to the surgical trajectory, potentially leading to numbness or dysesthesia of the upper lip. The anterior wall of the maxillary sinus was then removed while preserving the piriform aperture (Fig. 1c).

## Result

Five patients were identified. Two patients (trigeminal schwannoma and sphenoid sinus encephalocele) underwent EMMM alone (Table 1; Case 1,2), and the other three (large meningiomas extending to the ITF/PPF) underwent a combination of EMMM and DALMA (Table 1; Case 3–5). In the combined approach, the lesions extended as far as the medial border of the mandible, and the dual access points through the maxillary sinus minimized interference between the endoscope and instruments, achieving resection rates of 90%−98%. Postoperative complications were minimal. Two patients experienced permanent symptoms; one with slight facial sensory impairment noted in the patient with trigeminal schwannoma treated ETS with EMMM alone (Case 1) and one with slight localized numbness in the ipsilateral upper lip likely due to ASAN injury related to DALMA (Case 4).

**Table 1:** Summary of patients with skull base pathology treated with extended modified medial maxillectomy and direct approach to the anterior and lateral part of the maxillary sinus

Case	Age/Sex	Pathology	Location	Symptoms	Approach	Outcome	Complication
1	44/M	Trigeminal schwannoma	Meckel’s cave, PPF	Facial numbness	EMMM	GTR	Slightly worsening facial numbness
2	49/F	Idiopathic CSF leak	ITF	None	EMMM	Cure	None
3	85/F	Anaplastic meningioma	ITF, Meckel’s cave, PPF, sphenoid sinus	Trigeminal neuralgia, facial numbness	EMMM + DALMA	STR (90–98%), resolution of trigeminal neuralgia	None
4	84/F	Transitional meningioma	cavernous sinus, ethmoid sinus, ITF, Meckel’s cave, orbit, PPF, sphenoid sinus	Blind, ophthalmoplegia, proptosis	EMMM + DALMA	STR (90–98%)	Slight numbness in upper lip
5	35/F	Atypical meningioma	cavernous sinus, ITF, Meckel’s cave, orbit, PPF, sphenoid sinus	Oculomotor nerve palsy, facial numbness	EMMM + DALMA	STR (90–98%)	Transient abducens palsy

*CSF* Cerebrospinal fluid, *DALMA* Direct approach to the anterior and lateral part of the maxillary sinus, *EMMM* Extended modified medial maxillectomy, *F* female, *GTR* Gross total resection, *ITF* Infratemporal fossa, *M* Male, *PPF* Pterygopalatine fossa, *STR* Subtotal resection

**Fig. 2:**
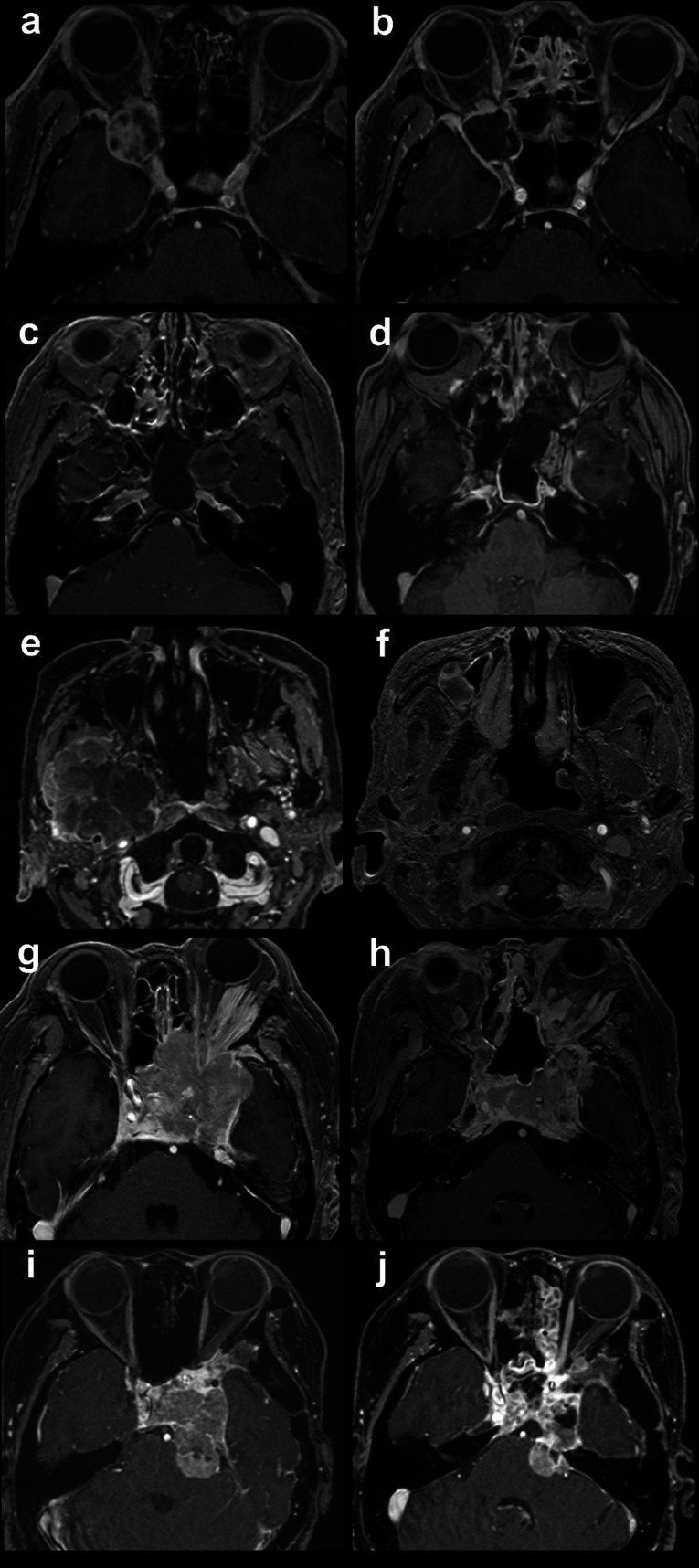
Preoperative images of all five patients are shown in the left column, and postoperative images in the right column. Case 1, a trigeminal schwannoma involving Meckel’s cave and pterygopalatine fossa (PPF) **(a),** achieved gross total resection using only route of endoscopic modified medial maxillectomy (EMMM) **(b)**. Case 2, an idiopathic cerebrospinal fluid leak in the infratemporal fossa (ITF) **(c)**, achieved cure using only route of EMMM **(d)**. Case 3, an anaplastic meningioma extending from the sphenoid to Meckel’s cave, PPF, and ITF** (e)**, achieved subtotal resection using combination of EMMM and DALMA **(f)**. Case 4, a transitional meningioma involving the cavernous sinus, ethmoid sinus, sphenoid bone, Meckel’s cave, orbit, PPF, and ITF **(g)**, achieved subtotal resection using EMMM and DALMA **(h)**. Case 5, an atypical meningioma involving the cavernous sinus, sphenoid bone, Meckel’s cave, orbit, PPF, and ITF **(i)**, achieved subtotal resection using EMMM and DALMA **(j)**

As a representative surgery, Case 3, a giant anaplastic meningioma, is presented in the accompanying video. The tumor extended superiorly from the sphenoid sinus to Meckel’s cave, with partial invasion into the PPF and extensive lateral extension into the ITF. Combining EMMM with DALMA enabled the introduction of a dedicated port for endoscopic visualization and a separate working corridor for surgical instruments through the medial wall of the maxillary sinus, thereby providing a wider lateral operative field. Figure 1 illustrates the preoperative surgical planning and the extent of bony removal in Case 3 with the combined EMMM-DALMA approach. Figure 2 presents Cases 1–5 in alphabetical order, with the left and right columns corresponding to pre- and postoperative images, respectively.

## Discussion

This study demonstrated the efficacy of combining two approaches, EMMM and DALMA, in ETS for lesions involving the ITF and PPF. The EMMM provides a straight surgical trajectory to these regions, proving to be a useful technique. However, in skull base surgery, where bimanual operation is often required, EMMM alone may limit access to extensive lesions. This limitation arises from the conflict between instruments and the endoscope when operating through the narrow space between the medially displaced inferior turbinate and the piriform aperture. The Caldwell-Luc approach is an established procedure to directly access to the maxillary sinus lesions in otorhinolaryngology. We acknowledge that approaches incorporating an external transmaxillary corridor, such as the Caldwell–Luc approach, may allow the use of larger conventional instruments with wider triangulation. However, in our experience, the combination of long-shaft instruments and internal corridor divergence between the EMMM and DALMA windows provided sufficient reach and hemostatic control for vascular tumors such as meningiomas without requiring an external incision. Thus, while nostril crowding cannot be completely eliminated, the internal geometric separation of the two corridors substantially reduces instrument conflict during deep lateral dissection [2, 7, 10, 11]. Based on a similar concept of a transmaxillary approach involving drilling of the anteromedial wall of the maxillary sinus, the Denker approach has been modified into an endoscopic technique to reduce invasiveness. However, its application in skull base neurosurgery has rarely been reported, particularly for tumors involving the ITF and PPF [5, 12–14, 21].

In ETS for skull base lesions, the potential advantages of multiportal techniques, which involve the separation of surgical instruments and the endoscopic port, have attracted significant academic interest. Cadaver studies comparing endoscopic endonasal, transantral transpterygoid, and lateral transorbital approaches have demonstrated that each corridor provides region-specific advantages in surgical freedom and area of exposure, with medial compartments better addressed through endonasal routes and lateral targets more effectively accessed through the other corridors; importantly, combining approaches significantly increases the total area of exposure [8, 15, 19]. Similarly, multiportal transnasal–transoral–transpharyngeal techniques have been shown to expand inferior and lateral reach while reducing instrument crowding through separation of visualization and working ports [23]. Clinical case series of transspatial retromaxillary tumors further illustrate that adding a transmaxillary window enables two-surgeon cooperation and improved maneuverability toward posterolateral extensions that would be difficult to reach through a single nasal corridor [6]. A multiportal technique combining EMMM with the Caldwell–Luc approach has also been reported specifically for tumors involving the ITF [11]. Collectively, these reports support a corridor-based concept in which complementary access routes are selected according to lesion location; however, these concepts require multiple incisions for improved surgical maneuverability and lateral access.

In this context, the DALMA provides direct access to more lateral regions of the PPF and ITF and functions effectively as a multiportal technique for bimanual maneuverabliy when combined with EMMM, likewise to less invasive endoscopic Denker approach. Notably, the addition of DALMA to EMMM creates dual entry routes into the maxillary sinus, reducing interference between the instruments and the endoscope and enabling resection of 90%–98% of the lesion volume. In addition, complications in this case series were minimal. Although ASAN injury and resultant numbness and dysesthesia of the upper lip may be an unavoidable risk when adding DALMA to EMMM [18, 20], our study found only one of three patients who experienced such a complication. While ASAN injury in DALMA may be minimal or even asymptomatic in some patients, it should be noted that the patients in this series already had preexisting trigeminal numbness, which may have masked a subclinical ASAN injury. In the trigeminal schwannoma case, mild postoperative facial hypoesthesia occurred, likely due to tumor involvement of the trigeminal nerve rather than the surgical corridor. Given the limited size of this preliminary series, the safety implications of reduced visualization during instrument entry should be interpreted cautiously. While we managed this trade-off by restricting the blind phase to the brief entry period and performing all critical maneuvers under continuous endoscopic visualization, further accumulation of cases is required to better characterize procedure-specific morbidity and to refine risk-mitigation strategies.

In neurosurgical practice, the combined use of EMMM and DALMA extends the lateral reach of endoscopic transnasal skull base approaches, offering a minimally invasive yet effective option for the management of complex lesions extending into the ITF and PPF, and representing a promising strategy for future skull base surgeries.

## Conclusion

The combination of EMMM and DALMA allows access to more lateral regions in the endoscopic transnasal skull base surgery for ITF and PPF lesions. Separation of the entry routes for the endoscope and instruments enhances maneuverability and facilitates exposure extending to the medial border of the mandible, without evident increase in morbidity in this preliminary series.

## Supplementary Information

Below is the link to the electronic supplementary material.

Supplementary file1 (MP4 445403 KB)

## Data Availability

No datasets were generated or analysed during the current study.
